# Activated Carbon Blended with Reduced Graphene Oxide Nanoflakes for Capacitive Deionization

**DOI:** 10.3390/nano11051090

**Published:** 2021-04-23

**Authors:** Gbenro Folaranmi, Mikhael Bechelany, Philippe Sistat, Marc Cretin, Francois Zaviska

**Affiliations:** Institut Européen des membranes, IEM, UMR-5635, Université de Montpellier, ENSCM, CNRS, Place Eugène Bataillon, CEDEX 5, 34095 Montpellier, France; gbenro.folaranmi@etu.umontpellier.fr (G.F.); philippe.sistat@umontpellier.fr (P.S.); marc.cretin@umontpellier.fr (M.C.)

**Keywords:** desalination, activated carbon electrode, reduced graphene oxide, composite electrodes

## Abstract

Capacitive deionization is a second-generation water desalination technology in which porous electrodes (activated carbon materials) are used to temporarily store ions. In this technology, porous carbon used as electrodes have inherent limitations, such as low electrical conductivity, low capacitance, etc., and, as such, optimization of electrode materials by rational design to obtain hybrid electrodes is key towards improvement in desalination performance. In this work, different compositions of mixture of reduced graphene oxide (RGO) and activated carbon (from 5 to 20 wt% RGO) have been prepared and tested as electrodes for brackish water desalination. The physico-chemical and electrochemical properties of the activated carbon (AC), reduced graphene oxide (RGO), and as-prepared electrodes (AC/RGO-x) were characterized by low-temperature nitrogen adsorption measurement, scanning electron microscope (SEM), X-ray diffraction (XRD), Raman spectroscopy, X-ray photoelectron spectroscopy (XPS), Fourier transform infra-red (FT-IR), cyclic voltammetry (CV), and electrochemical impedance spectroscopy (EIS). Among all the composite electrodes, AC/RGO-5 (RGO at 5 wt%) possessed the highest specific capacitance (74 F g^−1^) and the highest maximum salt adsorption capacity (mSAC) of 8.10 mg g^−1^ at an operating voltage ∆E = 1.4 V. This shows that this simple approach could offer a potential way of fabricating electrodes of accentuated carbon network of an improved electronic conductivity that’s much coveted in CDI technology.

## 1. Introduction

Unfavorable environmental factors such as water contamination, industrialization, climate change, etc., have led to a high demand in sourcing for alternative means of fresh water [1,2]. Available water technologies such as reverse osmosis (RO), multiple-effect distillation (MED), multi-stage flash distillation (MSFD) [3], etc., have proffered a long-standing solution to fresh water shortage by desalination seawater. This technology, for instance, RO, involves basic principle of abstracting solvent from solute under high pressure; as a result, it requires high energy consumption and it is liable to suffer from challenges such as membrane fouling and other limitations. Consequently, it is imperative to provide a simple, low cost, and environmentally friendly water desalination technology that can exploit surface water of low salt concentration (brackish water), such as against seawater.

Capacitive deionization (CDI) is an environmentally friendly brackish water desalination technology that temporarily and electrostatically separates and stores solvated ions by using porous materials as electrodes. An electric field is created when a potential difference is applied potential (as low as ΔE = 1.2 V) to electrostatically attract ions for temporary storage in the pores of the materials and subsequently get discharged as soon as ΔE is canceled or reversed [4,5].

Carbonaceous materials have always been the focus of attention as electrode materials in CDI because of the availability of cheap source of precursory material, i.e., biomass and their high surface area and porosity [6]. The material aspect of this technology is of utmost importance for optimization in order to achieve suitable or hybrid electrodes of high performance with respect to the aforementioned properties; thus, there have been relentless approaches towards improving this aspect [7,8,9,10].

Activated carbon has attracted attention because of its cheap cost and the fact that it can be easily prepared from readily available biomass has made it a focal point as a main electrode material in CDI. However, this cheap material is not without some challenges, such as low hydrophilicity, low electrical conductivity, etc. Thus, in order to overcome its challenges, AC surface chemistry is usually modified by various methods, such as oxidation with oxidizing agents [11], or combined with additives to prepare hybrid electrode materials of high performance [5].

Reduced graphene oxide (RGO) is made of single or more layers of graphite sheets of high electrical conductivity and has found application as a super-capacitor due to its high capacitance, surface area, and superior electrical conductivity [12]. Since its discovery, RGO has been independently used as a promising electrode material candidate or as an additive with other carbon-based materials in CDI [13]. As recently reported, advances in RGO nanoflakes has shown that methods of graphene oxide (GO) reduction to yield RGO nanoflakes could affect its textural properties. RGO synthesized by Li et al. [14] using the hydrazine reductive method on GO, yielded RGO possessing 222.01 m^2^ g^−1^ specific surface area with an electrosorptive performance of 1.35 mg g^−1^. Since desalination performance of carbon-based electrodes is dependent on properties such as the electroactive surface area, microstructure, porosity, etc., several attempts have been made to fabricate electrodes of a well-accentuated carbon network in order to forestall the challenges posed by the RGO method of synthesis [15]. However, RGO reportedly used for this purpose (as an additive) usually undergoes the use of toxic chemicals for synthesis and the need of pre-treatment steps in order to improve its performance. Such pre-treatments, for instance, involve thermal treatment, which is often time consuming. As a matter of diversity, we propose in this paper a simple and non-toxic reductive method of RGO synthesis by GO reduction using potassium hydroxide (KOH) and then subsequently used an additive to influence the electrical conductivity of commercial activated carbon (AC). The as-prepared RGO was combined with commercial AC without any pre-treatment step for desalination application using the CDI technique.

In the field of super-capacitors, RGO nanoflakes have found application as an additive to improve the electrical conductivity of electrochemically-active double-layer materials [16,17,18,19,20,21,22]. Chuanyin et al. [21], through hydrothermal and CVD processes, synthesized 3D flexible nitrogen-doped RGO (NRGO) and, thereafter, combined with carbon nanotubes to fabricate a flexibly hybrid super-capacitor of high specific capacitance and retention.

Thus, in the same way, we propose here a series of hybrid AC/RGO-x made by combining commercial AC with RGO nanoflakes produced by KOH reduction of graphene oxide (GO). The composites were synthesized and labeled as AC/RGO-x where x represents the percentage weight composition of RGO in the composite. (x = 5, 10, 15, and 20 wt% RGO). The electrochemical properties of the prepared electrodes (AC, AC/RGO-x) were analyzed by cyclic voltammetry (CV) and electrochemical impedance spectroscopy (EIS) after which the electrodes were tested in a laboratory-made CDI cell for desalination application. Results obtained were compared to the ones reported in the literature and our findings proved the interest of our approach for a prospective low-cost desalination.

## 2. Materials and Methods

### 2.1. Materials

Polyvinylidenefluoride (PVDF) (CAS no: 24937-79-9, Alfa Aesar, Erlenbachweg 2, Kandel, Germany), *N*-methyl-2-pyrrolidone (NMP) (CAS no 872-50-4, 99.7%, M.W 99.13 g/mol), hydrochloric acid (HCl) (CAS no: 7647-01-0, 37%), activated carbon (Supelco Analytical, CAS no: 7440-44-0), and graphite powder (CAS no: 7782-42-5) were supplied by Sigma Aldrich (Steinheim, Germany). Carbon black (CAS no: 1333-86-4) was supplied by Alfa Aesar (Steinheim, Germany). Graphite foil (0.35 mm thick) was supplied by RMC Remacon (Bad Säckingen, Germany).

Phosphoric acid (H_3_PO_4_), (CAS no: 7664-38-2, 85%) and potassium permanganate (KMnO_4_) (CAS no: 7722-64-7, 98%) were purchased from Alfa Aesar (Erlenbachweg 2, Germany). Sulfuric acid (H_2_SO_4_) was purchased from ACS Reagent (Belmont, CO, USA). Absolute ethanol (CAS no: 64-17-5, 99.8%) and hydrogen peroxide (CAS no 7722-84-1, 7732-18-5 30%) were purchased from VWR chemicals (Fontenay-sous-Bois, France). All chemicals were used as supplied.

### 2.2. Experimental Procedure

#### 2.2.1. Graphene Oxide Synthesis

GO was synthesized by modified Hummers method [23]. Briefly, 3 g of graphite powder and 18 g of potassium permanganate was gently added into a mixture of 40 mL of phosphoric acid and 360 mL of sulfuric acid. The solution was mildly stirred for 18 h at room temperature. After, the solution was poured onto ice (400 mL) with 3 mL of H_2_O_2_ then it was stopped after few minutes for homogeneity. The solution was centrifuged (4000 rpm for 10 min) to recover the sediment from the supernatant and the sediment was then washed multiple times following the procedure described elsewhere [24,25].

#### 2.2.2. Reduced Graphene Oxide Synthesis

A total of 3.7 g of as-prepared GO was mixed with 60 mL of 8 M KOH and sonicated for 2 h. After sonication, it was washed multiple times with distilled water under centrifugation and later dried in an oven at 80 °C for 2–4 h.

#### 2.2.3. Fabrication of Activated Carbon and AC/RGO-x Electrodes

AC electrode was prepared using activated carbon powder with specific surface area of 1031 m^2^ g^−^^1^. AC slurry was prepared as a suspension of activated carbon powder (3.20 g), carbon black (0.40 g) and PVDF (0.40 g) in 25 mL NMP. The mixture was stirred for 2 h and sonicated for 40 min to ensure homogeneity. The slurry was then deposited on a graphite sheet by spray coating (Air Brush Iwata, Yabukicho, Fukushima Prefecture, Japan) and then dried at 80 °C for about 1–3 h. Similarly, the as-prepared RGO (5, 10, 15, and 20 wt%) was added to specific mass of AC containing PVDF and the mixture was agitated for 2 h in 25 mL NMP. The aforementioned procedure was then followed to obtain the composites AC/RGO-x.

### 2.3. Physical Characterization

Scanning electron microscopy (SEM) was used to analyze the structure of the samples (FESEM, Hitachi S4800, Tokyo Japan). The structural properties were studied by Raman spectroscopy (HORIBA Xplora, Minami-ku, Kyoto, Japan) and X-ray diffractometer (XRD Pan Analytical X’pert Phillips, Lelyweg, EA Almelo Netherlands) with Cu Kα radiation λ = 0.15406 nm at 40 kV and 30 mA). X-ray photoelectron spectroscopy (XPS ESCALAB 250 Thermo Electron, Strasbourg, France) was also used for moieties identification. For the XPS analysis, the excitation source is the monochromatic source Al line Kα with a photo energy observed at 1486.6 eV. The analyzed surface has a diameter of 500 µm. The photoelectron spectra are calibrated in terms of bond energy with respect to the energy of the C=C component of carbon C1 s at 284.4 eV. Surface area was obtained by using N_2_ adsorption/desorption at 77 K. S_BET_ was the specific surface area calculated by the Brunauer–Emmett–Teller (BET) method (Micromeritics 2020 ASAP, Merignac, France). Vt was the total pore volume calculated from the amount adsorbed at a relative pressure (P/P°) of 0.99, V_meso_ was the mesopore volume calculated by the Barrett–Joyner–Halenda (BJH) model. UV–VIS spectrophotometry measurement was conducted using UV 2401PC SHIMADZU Corporation, Tokyo, Japan) in order to ascertain the shift in the wavelength of absorption and FTIR measurement was carried out using Thermo Nicolet Nexus 4700 ATR-FTIR (Thermo Electron Corporation, Montréal-Est, QC, Canada) in order to verify the functional finger prints of the samples.

#### 2.3.1. UV–VIS Spectrophotometry Measurement

Briefly, dilute dispersion of GO and RGO in water were sonicated for 1 h. RGO dispersion was further sonicated for additional 1 h due to its low solubility in water and, thereafter, the supernatant of the two products (GO and RGO) were taken and injected in a cuvette of 4.5 cm path length which was thereafter placed in UV–VIS spectrophotometer for absorbance measurement and UV recording.

#### 2.3.2. FTIR Measurement

Briefly for the FTIR measurement, a few milligram of the powdered sample was placed on a doted red spot. Thereafter, it was made to have contact with the ATR detector by gentle pressing and the infrared transmittance was monitored and recorded on a computer using OMNIC software.

#### 2.3.3. Electron Mobility Measurement

The four-point probe measurement was done following the procedure: Briefly, the material (AC, AC/RGO-x), deposited on graphite with an area of 1 cm^2^, was placed on a white plastic sample support. Before this, the difference in thickness of the graphite was measured before and after sample deposition in order to ascertain the thickness of the sample deposited. Thereafter, the four probes were set and fixed at a particular position to maintain regular contact when placed upon the sample support and the hall effect software was launched in order to take the resistivity values. After this, electron mobility measurement was carried out by replacing the four probes with hall probe (a giant bar made to focus on the sample support for few seconds approximately 30–40 s) to create magnetic field on the sample. As this is going on, the corresponding current and Hall voltage are recorded by the software, after which the electron mobility value is displayed.

### 2.4. Electrochemical Characterization

The cyclic voltammetry (CV) measurements of the prepared materials were performed with Origalys Potentiostat (OGF01A, Origalys Electrochem SAS, Les Verchères 2, Rillieux-la-Pape, France) with a three-electrode system at an operating window from −0.4 to 0.6 V vs. ref in 0.5 M NaCl electrolyte. The carbon electrode specimen with an exposed surface area of 1 cm^2^, platinum rod and 3 M KCl, Ag/AgCl electrode were used as the working electrode, counter electrode, and reference electrode, respectively. The double-layer capacitance C_DL_ can be determined from Equation (1) by plotting the charging and discharging currents as a function of scan rate from cyclic voltammetry measurements. The double-layer capacitance of the system is the average of the absolute value of the slope of the linear plot of charging and discharging currents fit to the data and Csp is the specific capacitance (F g^−^^1^) determined by considering the mass (g) of the active material on an electrode surface of 1 cm^2^ as shown in Equations (1) and (2), respectively:

i = υ C_DL_ (1)

Csp = C_DL_/m (2)

where i is the double-layer charging current, υ the scan rate and C_DL_, the electrochemical double-layer capacitance, and m is the deposited mass of the active material.

Electrochemical impedance spectroscopy (EIS) was performed using the above set-up with an Origalys Potentiostat (OGF01A, Origalys Electrochem SAS, Les Verchères 2, Rillieux-la-Pape, France) at an operating frequency of 5000 kHz to 10 MHz at a sine wave of 5 mV. The galvanostatic charge and discharge (GCD) of the electrodes were also carried out following the above aforementioned set-up (as explained in CV) but at a constant current density of 0.1 A. g^−^^1^ within a potential window of 0–2.5 V with an Origalys Potentiostat (OGF01A, Origalys Electrochem SAS, Les Verchères 2, France). The Hall effect for electron mobility was performed on four probes system using a Bio-Rad HL5500PC (Hall Polaron, Germany).

### 2.5. Capacitive Deionization Measurement

**Figure 1:**
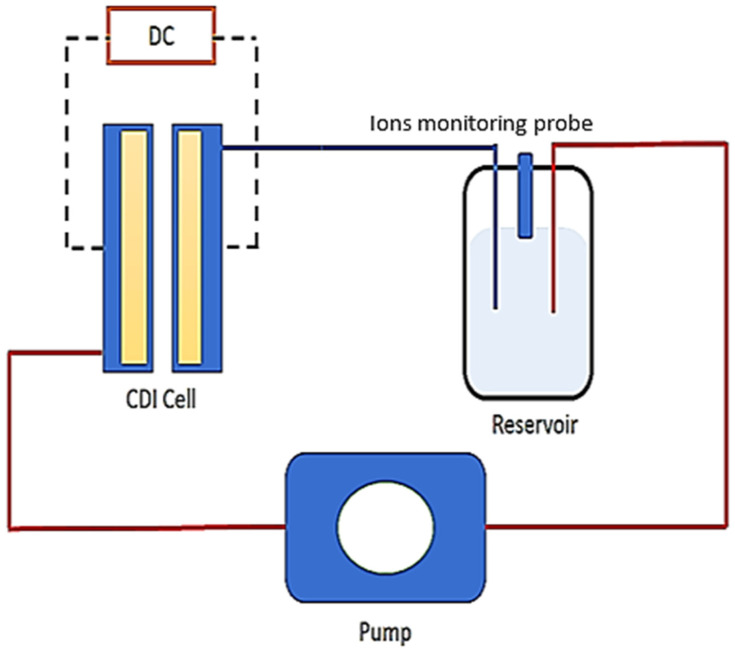
Schematic diagram of CDI experiment used in this study.

An electrosorption test (desalination) was performed in a batch mode system at constant voltage. Figure 1 shows the schematic representation of the experiment while Table S1 presents the solid electrode formulation of this experiment. It consists of a CDI unit cell, a peristaltic pump, a power source (Origalys potentiostat PST OGF01A, Origalys Electrochem SAS, Les Verchères 2, Rillieux-la-Pape, France) and a conductivity probe monitor (Hanna Instruments SRL, Nusfalau, Str. Hanna, 457260 Jud. Salaj, Romania) inserted in a tank containing the influent. In this work, a homemade CDI cell consisting of a stack of plexiglass that is coated with graphite material as current collectors into which the electrode sheets are inserted was used. The electrodes are separated by a non-electrically conductive spacer (0.99 mm thick) which allows passage of salt solution (influent). The electrode materials are directly attached to the current collectors which are subsequently connected to an external power source (potentiostat). The CDI electrodes have an area of 60 cm^2^ and for each batch mode run, the saline solution was continuously pumped into the CDI cell at a constant rate of 25 mL min^−1^. The feed solution was a simulated brackish solution of 1200 mg L^−^^1^ of NaCl. The CDI tests were conducted at potential differences of ΔE = 1.4 V at a constant flow rate of 25 mL min^−1^. The difference in ionic conductivity was monitored continuously by a conductivity meter.

In the present work, the CDI performance indicators used to govern the performance of our cell are defined by the following parameters below:

Maximum salt adsorption capacity mSAC, Q (mg g^−^^1^) is defined by the mass of salt removed or abstracted to the deposited total mass of electrodes including binder (PVDF) and addictive (RGO) and it is expressed by Equation (3):

(3) $$Q=\;\frac{\left(Co-Ce\right)V}{m}$$

where **C_0_** and **Ce** are the initial and equilibrium concentration (mg L^−1^), respectively, **V** is the volume of the solution (L), and m is the total mass of the deposited electrode (g).

Average salt adsorption rate ASAR (mg g^−^^1^ min^−^^1^) is defined as the ratio of maximum salt adsorbed to the time of adsorption. It is related by Equation (4):

(4) $$\mathrm{ASAR}=\frac{Q}{t}$$

where **Q** is in mg g^−^^1^ and **t** is the time in minute.

Salt adsorption capacity, SAC (mg cm^−^^2^) is defined as the mass of salt adsorbed per the surface of electrodes and it is expressed by Equation (5):

(5) $$\mathrm{SAC}=\;\frac{\left(Co-Ce\right)V}{A}$$

**A** is the surface area of the electrode in cm^2^, i.e., 60 cm^2^ in our case.

Charge efficiency (CE) which relates to the ratio of salt adsorbed to the quantity of charge passed into the system was calculated by Equation (6):

(6) $$\mathrm{CE}=\frac{z\;\left(Co-Ce\right)\;V\;F}{⟆Idt}$$

where **z** is the equivalent charge of the ions, **V** is the volume of the solution (L), **F** is the Faradaic constant and ⟆**I***d***t** is the integrated quantity of charge passed to the system as a function of time.

## 3. Results

### 3.1. Morphology Properties of Precursors

The morphology of AC and the composites containing RGO was examined by a field emission scanning electron microscope (FESEM). The images are given in the supplementary material Figure S1a–f. Commercial AC shows an indefinite shape with no smooth surface (hill-like surface). RGO observations prove that the rough surface of the RGO sheet tend to stack or agglomerate together showing very limited porosity and compacted structure. On the other hand, all the composites containing RGO show no significant difference in their morphology when compared to that of the pristine AC and no significant evolution concerning the texture of the materials can be ascertained by SEM.

### 3.2. Structural Properties of Precursors

**Figure 2:**
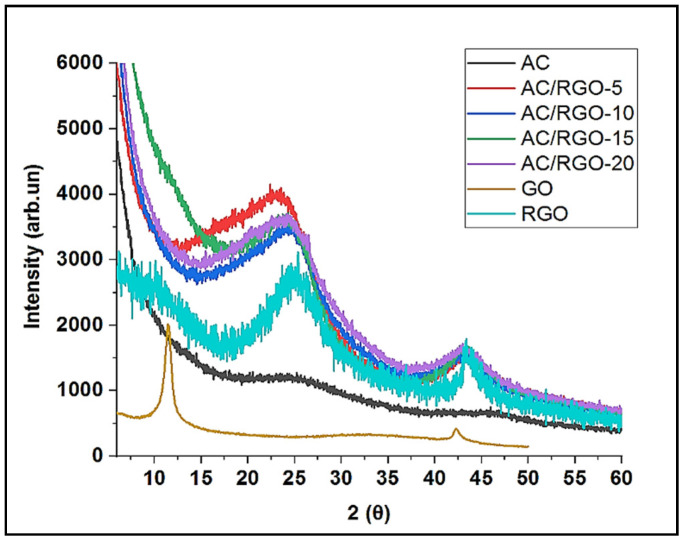
XRD patterns of AC, GO, RGO, and AC/RGO-x (where x = 5, 10, 15, and 20 wt% RGO)**.**

Structural properties were first investigated by X-ray diffraction. XRD patterns of GO, RGO and AC/RGO-x are shown in Figure 2. The XRD peak of graphene oxide was observed at 2 θ = 10.2° and a little sharp peak was also observed at 2 θ = 43°; showing a turbostratic disorder of GO due to its incomplete oxidation [26] while after chemical reduction, a little broad peak at 2 θ = 23.5° was observed in RGO indicating a layer-to-layer sheet of RGO [27]. Using Bragg’s law (nλ = 2dsin θ), where n is 1, λ is X-ray diffraction (1.541 A°), θ is the angle of diffraction in degree and d is the inter-planar or layer spacing between graphite layers. the inter-layer spacing, i.e., the distance between the adjacent sheets or layers of GO was calculated to be 0.9 nm and RGO was 0.36 nm, respectively. This shows that after chemical reduction using KOH, there is a decrease in the inter-layer spacing in the graphitic layer of RGO due to the removal of most oxygenated functional groups in GO [28]. No significant difference was observed in the inter-layer spacing of pristine AC and its composites (*d*002 of 0.36 nm). The XRD pattern of AC/RGO-x composites exhibited combined characteristics with broad peaks at 2θ = 23.5–25.9° (002 plane) and 44.5° (10/101 plane). The 002 planes correspond to the graphitization of the organic component and nano-crystalline structure of the matrix, while the 10/101 plane reveals the formation of a 2D graphitic-like structure [28].

**Figure 3:**
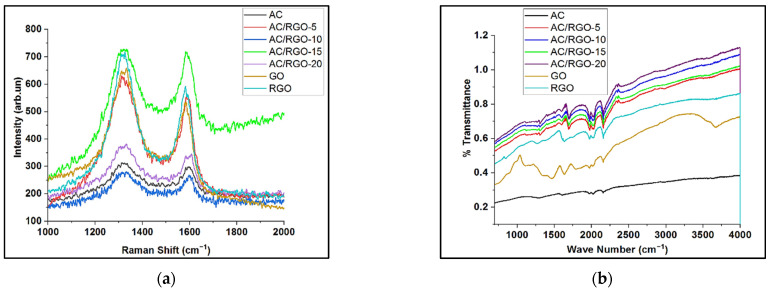
(**a**) Raman spectra of precursors and AC/RGO-x composites; (**b**) FTIR spectra of precursors and AC/RGO-x (where x = 5, 10, 15, and 20 wt% RGO) composite electrodes.

Raman spectroscopy analysis was done to understand the level of defect and disorder in the materials. All of our materials possess characteristic features of graphitic carbon with Raman shift at 1350 cm^−^^1^ and 1590 cm^−^^1^ corresponding to D and G bands, respectively, as shown in Figure 3a. D band arises from a defect that is based on structural edge effect due to breathing mode of rings while D band relates to the ordered structure of graphite crystals [25]. From the Raman spectra, GO possessed broad D band due to the size reduction of sp^2^ domains that is caused by distortion during oxidation [29]. The intensity ratio I_D_/I_G_ is an important parameter used to observe the level of disorder in carbonaceous materials. In our materials, no significant increase or decrease in defects of the composites in comparison to pristine AC was observed as shown in Table S2.

Using FTIR, the identification of functional groups presents in GO, RGO, AC, and its composites were performed, as made evident in Figure 3b. The peak at 3700 cm^−^^1^ is associated with the –OH stretching of alcohol or phenol [30] which was significantly reduced in RGO on comparison with GO. The peaks present at 1720 cm^−^^1^ is due to C=O of carbonyl group and it is seen to be most pronounced in GO, which is abundantly rich in oxygenated functional groups and also more obvious in all the composites than in AC. Additionally, the peak at 1382 cm^−^^1^ is due to C–H stretching [30].

Thermogravimetric analysis (TGA) that reveals the stability of the precursors (GO and RGO) and results showing the effectiveness of the chemical reduction by XPS are given in Supplementary Information Figures S2 and S3a–c, respectively. In Figure S4, the reduction of GO and RGO is further confirmed by UV–VIS absorption spectroscopy with GO having an absorption maximum at 205 nm while, after reduction to RGO, this absorption maximum is red-shifted to 214 nm owing to the restoration of sp^2^ carbon atoms [31].

### 3.3. Textural Properties of Electrodes

**Figure 4:**
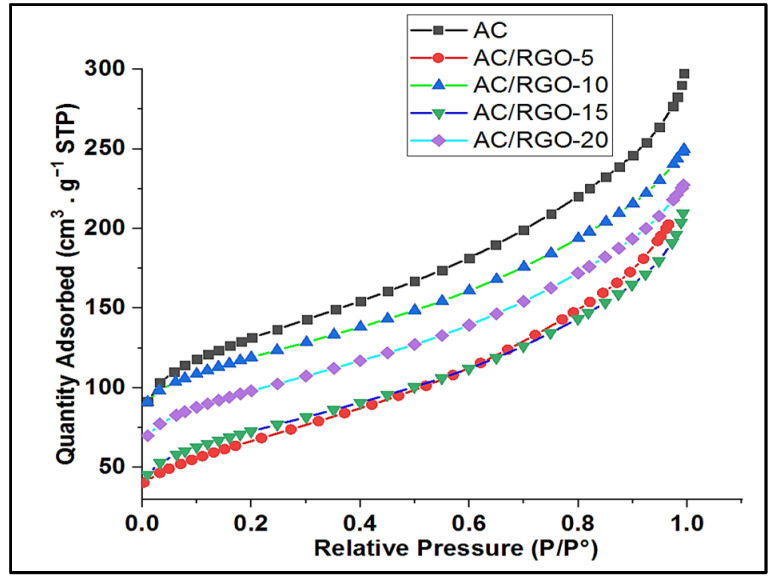
Isotherm linear plot of AC, and AC/RGO-x (where x = 5, 10, 15, and 20 wt% RGO).

In the present work, AC and AC/RGO-x (x = 5, 10, 15, and 20 wt% of RGO) electrodes were synthesized for usage as active phases of CDI, and then there is a great interest to determine and compare their textural properties. Figure 4 gives the adsorption isotherms used to determine the main parameters expressed in Table 1. The inclusion of PVDF in electrode synthesis possibly leads to a significant decrease in the specific surface area of AC from 1031 m^2^ g^−^^1^ (initial value of AC used as precursor) to 474 m^2^ g^−^^1^ for the electrode (Table 2). Also, the low specific surface area (SSA) or S_BET_ of GO and RGO is due to the dense restacking of GO sheets as a result of van der Waal force between the layers and this factor consequently have negative effect on the effective surface area of RGO [32].

From Figure 4, AC and AC/RGO-x isotherms are similar to each other with a characteristic mesporous layer due to the sharp adsorption uptake between 0.1 and 1.0 relative pressure (P/P°) indicating a type II isotherm based on IUPAC classification [33]. Additionally, based on the isotherm curves of the samples, a non-mutual curve arrangement was observed as shown in Figure 4; this could be due to additive effect on the textural properties of AC and this effect, is independent on additive ratio. The textural parameters of AC and AC/RGO-x (different electrode values) collected are summarized in Table 2. It is obvious that, regardless of RGO content in the composite, no significant effect on textural properties were observed. The synthesized RGO has a low specific surface area and pore volume indicating its low porosity; a result in line with what is reported by Wenhua et al. [34]. The addition of additives at different proportion however do not have a significant reduction in the specific surface area of the pristine electrode, indicating good interaction that led to preservation of the specific surface area of AC by RGO sheets. Our result is in agreement with a study reported by Choi et al. [35].

### 3.4. Electrochemical Property

**Figure 5:**
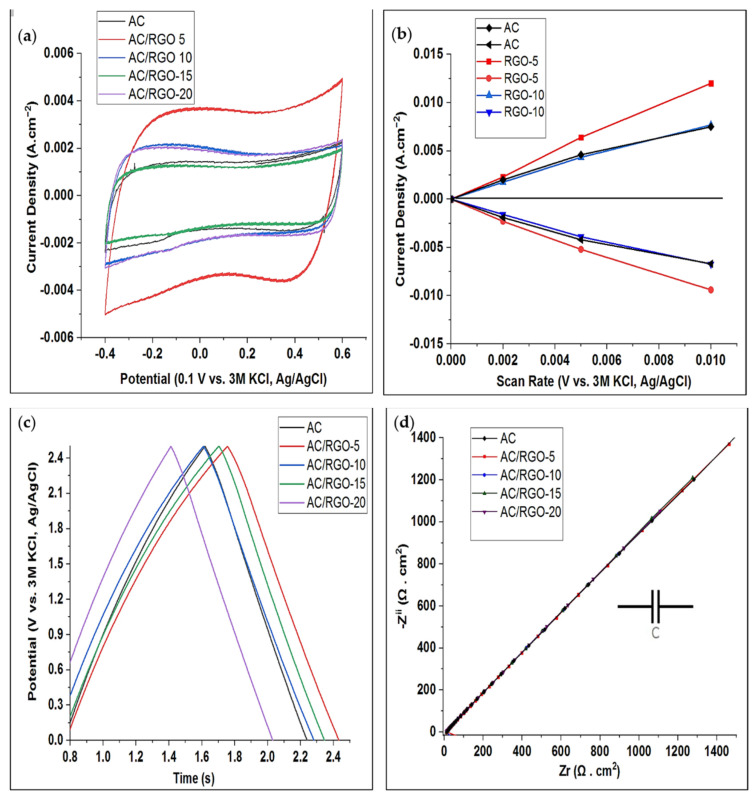
Cyclic voltammogram of (**a**) AC and AC/RGO-x (where x = 5, 10, 15, and 20 wt% RGO) at 2 mV s^−1^ s^−1^ (**b**) Plots of charging current (above 0 A cm^−2^) and discharging currents (below A cm^−^^2^) against the scan rate (V.s) for C_DL_ determination (**c**) Galvanostat charge-discharge (GCD) of AC and AC/RGO-x at 0.1 A g^−^^1^ and (**d**) Nyquist plot of AC and AC/RGO-x.

Electrochemical behavior of the pristine AC and composite electrodes were carried out using cyclic voltammetry (CV) at potential window of −0.4–0.6 V (to ensure electrochemical stability of the electrolyte and prevent water splitting, i.e., oxygen and hydrogen evolution) and different scan rates in order to investigate the influence of the additive at different ratio. The experiment was conducted in 0.5 M NaCl aqueous solution. CV is an important parameter carried out to understand the adsorption behavior and capacitive nature of carbon-based materials. In Figure 5a, the CV curves at low scan rate (2 mVs^−1^) of the electrodes show symmetrical almost rectangular shapes with no definite or obvious redox peaks indicating the capacitive behavior of the electrodes. This phenomenon is possibly due to the fact that at this scan rate, ions have enough time to migrate into the pores of the materials thus exhibiting good capacitive behavior. On comparison with pristine AC in Figure 5a, it is obvious that AC/RGO-5 exhibited largest area and current response. This shows that little proportion of RGO at 5 wt% is enough to improve the capacitive behavior of AC. The double layer capacitance was calculated from CV curves at different scan rates (2–10 mVs^−1^) by taking into consideration open circuit potential at 0.1 V vs. 3 M KCl, Ag/AgCl while specific capacitance of the materials was calculated by dividing the calculated double layer capacitance by the deposited mass of the electrode Equation (2). Figure 5b shows the linear plot of AC and the composites in which the positive and negative capacitive currents taken at 0.1 V vs. 3 M KCl, Ag/AgCl (OCP) is plotted against a range of scan rates (2–10 mV s^−1^). The EDL capacitance (C_DL_) is taken as the average of the absolute value of the slope of the linear plot of the positive and negative capacitive current fit to the data according to Equation (1). The calculated EDL capacitance (C_DL_) of all the electrodes is presented in Table 2. From the table, it can be seen that the dominating contribution of RGO at the ratio of 5 wt% is more prominent in the electrochemical performance (highest C_DL_) of AC/RGO-5 possibly due to the fact that the dopant level at this ratio offers a synergetic effect by creating a well interconnected network structure within the RGO sheets thus providing conductive bridge for AC that enables faster transport of ions to its pores. Although the specific surface area (SSA) of AC/RGO-5 is lower in comparison to other composites, yet its capacitance is higher; showing that SSA is not the only contributing factor for higher electrochemical performance.

Galvano-static charge-discharge (GCD) was also performed to ascertain the reversibility nature of the electrodes. As shown in Figure 5c, the symmetrical triangular shapes of GCD without any form of deviation show clearly that the storage mechanism is dominantly EDL in nature and that all the electrodes are reversible. Additionally, the largest size of AC/RGO-5 under the curve corresponds or indicates its high specific capacitance.

The Nyquist plots of the EIS data of the materials are presented in Figure 5d. In EIS, solution resistance (Rs) is the beginning of the semicircle line at Z^i^ (left intercept of the real axis) and it is the resistance of the solution or electrolyte in use. The internal or intrinsic resistance (Rp) of the electrode (capacitor) marks the end or termination of the semicircle (right intercept at Zi real axis). Warburg diffusion at middle frequency region relates to the ease of diffusion of ions into the pores of the electrode while the capacitive behavior of the material is represented at low frequency range. As shown in Figure 5d, our materials exhibited capacitive behavior at low frequency range as no semi-circle was observed. As seen in Figure S5a, at the lower frequency range, AC/RGO-5 possessed lowest resistivity, thus leading to an efficient ions transport and faster diffusion into its pores, hence its highest capacitive behavior.

Figure S5b revealed the capacitive reactance variation of our samples. We believe that capacitive reactance of our electrodes became dominant in the low-frequency range as ions could migrate deeply to the pores (intrapores) of the electrodes and based on these results, AC/RGO-5 seems to be significantly favorable with this phenomenon [36]. From the EIS equivalent circuit fitting, AC/RGO-5 has the highest theoretical capacitance as shown in Figure S5c.

In order to further understand the effect of the additive, electron mobility behavior of the electrodes was studied on a four-probe system and, in all cases, AC/RGO-5 possessed an overall electron mobility characteristic. The electron mobility values of AC, AC/RGO-5, AC/RGO-10, AC/RGO-15, and AC/RGO-20 are 48, 55.5, 44.10, 24.40, and 23.70 cm^2^/(V.s) respectively. According to our result, AC/RGO-5 possessed a significant difference among its counterparts thus we opine that the dominating factor accounting to this electrochemical behavior might be due to the availability of electroactive species as a result of the improved interconnectivity network structures at this ratio. Our result is in agreement with the report of Zhi et al. [37].

### 3.5. Desalination Performance

**Figure 6:**
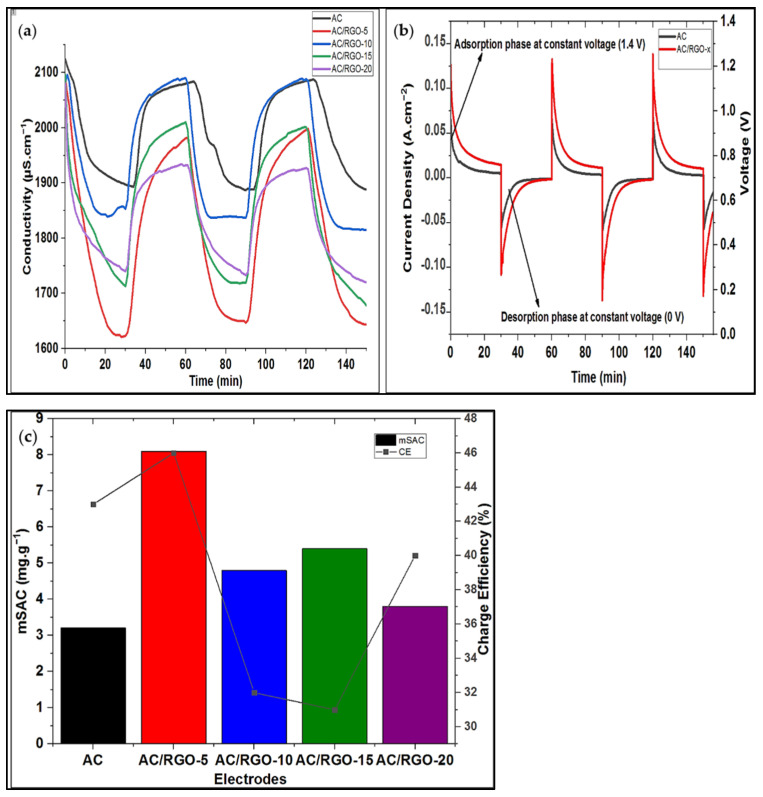
(**a**) Electrosorption curves of AC and AC/RGO-x at 1200 mg. L^−^^1^ NaCl solution under applied voltage of 1.4 V; (**b**) Current response of AC and AC/RGO-x (where x = 5 wt% RGO) at 1.4 V; (**c**) maximum salt adsorption (mSAC) and charge efficiency of AC and AC/RGO-x electrodes.

It is well known that desalination/electrosorption performances are not necessarily dependent on the specific surface area of carbon electrodes as other factors, such as porosity and electrical properties of the electrode materials, also contribute to this factor. It has been shown previously that the addition of a small amount of RGO to conventional AC improve significantly the electrochemical properties of the electrode in terms of capacitance, (74 F/g), and electron mobility (electrical properties). In order to confirm and make a link between the electrochemical properties and electrosorption, desalination experiments were performed in a closed loop system with saline solution of 1200 mg L^−^^1^ NaCl at a constant flow rate of 25 mL min^−^^1^ and at a cell potential ΔE = 1.4 V for 30 min per cycle using the different AC/RGO electrode ratio. At the end of each charging cycle (adsorption), the cell potential is maintained at zero voltage during discharging of ions (desorption). Conductivity values were recorded all along the adsorption and desorption phases as well as current intensity values Figure 6a,b. From these figures, performance indicators, such as mSAC and CE, can be extracted in order to evaluate and compare the electrosorption behavior of the pristine AC and its composite AC/RGO-x electrodes Figure 6c.

From Figure 6a, notable sharp drops of the initial concentration of the feed solution is made evident due to creation of electric field that led to fast migration of salt ions by electrostatic attraction into their different polarized electrodes during adsorption phase. It can observed that AC/RGO-x composites showed sharper drops in comparison with pristine AC while AC/RGO-5, among all its counterpart, exhibited the sharpest drop; a behavior that is expected due to its excellent electrochemical capacitive nature (fast-track ion adsorption on the electrode surface; stable EDL formation that enables ions storage) and in all cases, it exhibited the highest maximum salt adsorption capacity and charge efficiency as shown in Figure 6c (The fast ions mobility leading to quick salt extraction in AC/RGO-5 accounts for its highest charge efficiency). The CDI performance indicators (as summarized in Table 3) calculated from the evolution of Figure 6a,b using the Equations (3)–(6) proved that our strategy through this simple RGO synthesis and subsequent chemical agitation showed the obtained condition necessary for optimization of our electrodes for desalination. Lower desalination features of other composites AC/RGO-x (x > 5.0 wt% RGO) at high percentage could be due to the fact that at this ratio, the dense restacking of the RGO sheets which are closely stacked (agglomerated) must have significantly affected the diffusion pathways for the electrolyte ions into the EDLC, thus, decreasing the capacitive properties of the electrodes [38,39].

As presented in Table 4, AC/RGO composites of high specific capacitance using different approaches of RGO synthesis as against the method adopted by our group is found in the literature [40,41,42,43,44]. Undoubtedly, the method of synthesis and thermal pre-treatment affect functionalities (functional groups, textural and electrochemical properties) of carbon-based materials [34]. Using thermally pre-treated AC, Lanshu et al. [40] reported electrochemical studies of free-standing AC/RGO composites with an outstanding specific capacitance of 207 F g^−^^1^. Qiang et al. [41] synthesized a composite of ACF/RGO by electrospinning method and obtained specific capacitance of 193 F g^−^^1^ at the scan rate of 5 mV s^−^^1^. Similar reports have been made by Haibo et al. [42] and Xin et al. [43]. Interestingly, Xin et al. [43] reported a low electrosorption of 2.99 mg g^−^^1^ which is lower than the electrosorption reported for our composites thus showing the efficacy of our method which afforded a simple, cheap and less corrosive means of obtaining RGO as a dopant for commercial AC without any pretreatment to obtain a potential EDL hybrid electrode material. Though the reductive mechanism of GO to RGO with KOH is still unknown in literature but according to Xiaobin et al. [45], the reduction of graphene oxide under alkaline condition seems to be direct reversal of the oxidation process of graphite exfoliation under strong acidic condition.

## 4. Conclusions

In summary, a series of AC/RGO-x composite electrodes were made by simple blending process without any pretreatment step. RGO was synthesized by GO dispersion in solution of KOH. The resulting RGO was combined with commercial AC at different weight ratio and tested for desalination. Among all the composites, the electrode with 5 wt% of RGO (AC/RGO-5) exhibited an overall electrochemical behavior with double layer capacitance of 0.823 F cm^−2^ and maximum salt adsorption of 8.10 mg g^−1^ at operating potential window of ΔE = 1.4 V in 30 min in comparison with pristine AC with maximum salt adsorption of 3.20 mg g^−1^ at the same condition. Additionally, electrosorption performance and charge efficiency results of AC/RGO-5 show that the addition of RGO at this ratio is beneficial for a conducting network structure in AC, thus, giving rise to electroactive species that improves the capacitive nature of the composite electrode. In conclusion, this simple approach of material optimization can pave a new way of fabricating potential electrodes of high performance in CDI technique.

## Figures and Tables

**Table 1:** Textural parameters of pure AC, AC electrode, and AC/RGO-x (where x = 5, 10, 15, and 20 wt% RGO) electrodes and the additive.

Sample	V_t_ (cm^3^g^−1^)	S_BET_ (m^2^g^−1^)	V_MESO_ (cm^3^g^−1^)
Pure AC	0.82	1031.03	0.55
AC electrode	0.46	474.62	0.40
GO	0.01	5.48	0.01
RGO	0.03	16.37	0.06
AC/RGO-5	0.41	374.37	0.30
AC/RGO-10	0.43	473.42	0.38
AC/RGO-15	0.40	434.12	0.33
AC/RGO-20	0.45	480.73	0.39

**Table 2:** Calculated double layer capacitance (C_DL_) and specific capacitance (C_Specific_) for AC and AC/RGO-x electrodes.

	AC	AC/RGO-5	AC/RGO-10	AC/RGO-15	AC/RGO-20
C_DL_ (F cm^−2^)	0.29	0.89	0.74	0.49	0.65
C_Specific_ (F g^−1^)	32	74	48	29	50

**Table 3:** CDI operating performance matrices of this experiment at all operating conditions.

	mSAC (mg g^−1^)	SAC (mg cm^−1^)	ASAR (mg g^−1^ min^−1^)	CE (%)
AC	3.20	0.08	0.10	43
AC/RGO-5	8.10	0.23	0.27	46
AC/RGO-10	4.80	0.14	0.16	32
AC/RGO-15	5.40	0.17	0.18	31
AC/RGO-20	6.46	0.11	0.13	40

**Table 4:** Advances in electrosorption of AC/RGO composite electrodes in CDI.

Electrode Material	Synthesis Method	Applied Voltage ΔE (V)	[NaCl]° (mg L^−1^)	C_Specific_ (F g^−1^)	mSAC (mg g^−1^)	Ref
AC/RGO	Vacuum filtration process and *Th.T under N_2_	-		207	-	[40]
CNFs/RGO	Electrospinning and *Th.T under CO_2_	0.4–1.60	400	256	7.20	[41]
OAC/RGO	Oxidation/simple doping and *Th.T under N_2_	1.2–2.0	25	181	0.81	[42]
CNF/RGO	Electrospinning and *Th.T under N_2_	2.0	400	108	2.99	[43]
AC/FRGO	Sono-assembly and *Th.T under N_2_	1.80	117	27.9	12.58	[44]
NRGO-CNFs	Electrospinning and *Th.T under N_2_	1.20	100	337.85	3.91	[46]
RGO-CNFs	Electrospinning and *Th.T under N_2_	1.20	-	264.32	3.60	[46]
NC/RGO	Polymer templated method and *Th.T under N_2_	1.2	589	137.26	17.52	[47]
RGO/MC	Polymer templated method and *Th.T under N_2_	2.0	40	52.15	0.73	[48]
RGO/HCS	-	1.6	-	-	2.3	[49]
RGO/HPC	Dual template strategy and *Th.T under N_2_	1.2	25	80.34	6.18	[50]
RGO/CNTs/AC	Chemical mixing method	1.2	-	93.50	2.30	[51]
AC/RGO-5	Simple chemical method without *Th.T	1.4	1200	74	8.10	This work

*Th.T: Thermal treatment, FRGO: Functionalized reduced graphene oxide, CNF: Carbon nanofibers, OAC: Oxidized activated carbon, NC: Nitrogen-doped carbon, MC: Mesoporous carbon, HCS: Hollow carbon spheres, HPC: Highly-porous carbon, CNT: Carbon nanotubes.

## Supplementary Materials

The following are available online at https://www.mdpi.com/article/10.3390/nano11051090/s1, Figure S1: Field emission scanning electron microscope (FESEM) images of (a) AC (b) AC/RGO-5 (c) AC/RGO-10 (d) AC/RGO-15 (e) AC/RGO-20 and (f) RGO electrodes; Figure S2. Thermogravimetric curves of GO and RGO; Figure S3. Deconvoluted XPS spectra of C1s of (a) and (b) GO and RGO respectively (c) XPS whole survey of pristine GO and RGO; Figure S4. UV-Vis absorption spectroscopy shift of GO and RGO; Figure S5. (a) Bode (b) Capacitive reactance plot of AC and AC/RGO-x as a function of frequency and (c) Histogram plot of theoretical capacitance of AC and AC/RGO-x from equivalent circuit fitting; Table S1. Solid electrode composition.
