# Supplementary material for: Activated Carbon Blended with Reduced Graphene Oxide Nanoflakes for Capacitive Deionization
Source: Nanomaterials (Basel). 2021 Apr 23;11(5):1090. doi: 10.3390/nano11051090 (PMC8144981; doi:10.3390/nano11051090)
Supplement: Supplementary file 1 [file nanomaterials-11-01090-s001.zip › nanomaterials-1152315-supplementary.pdf]

nanomaterials

MDPI

Supplementary Materials

Activated Carbon Blended with Reduced Graphene Oxide Nanoflakes for Capacitive Deionization

Gbenro Folaranmi, Mikhael Bechelany *, Philippe Sistat, Marc Cretin, and Francois Zaviska *

Institut Européen des Membranes, IEM, UMR-5635, Université de Montpellier, ENSCM, CNRS, Place Eugène-Bataillon, Cedex 5, 34095 Montpellier, France; gbenro.folaranmi@etu.umontpellier.fr (G.F.); philippe.sistat@umontpellier.fr (P.S.); marc.cretin@umontpellier.fr (M.C.)

* Correspondence: author: Mikhael.bechelany@umontpellier.fr (M.B.) François.zaviska@umontpellier.fr (F.Z.)

1. Result

1.1. Morphology

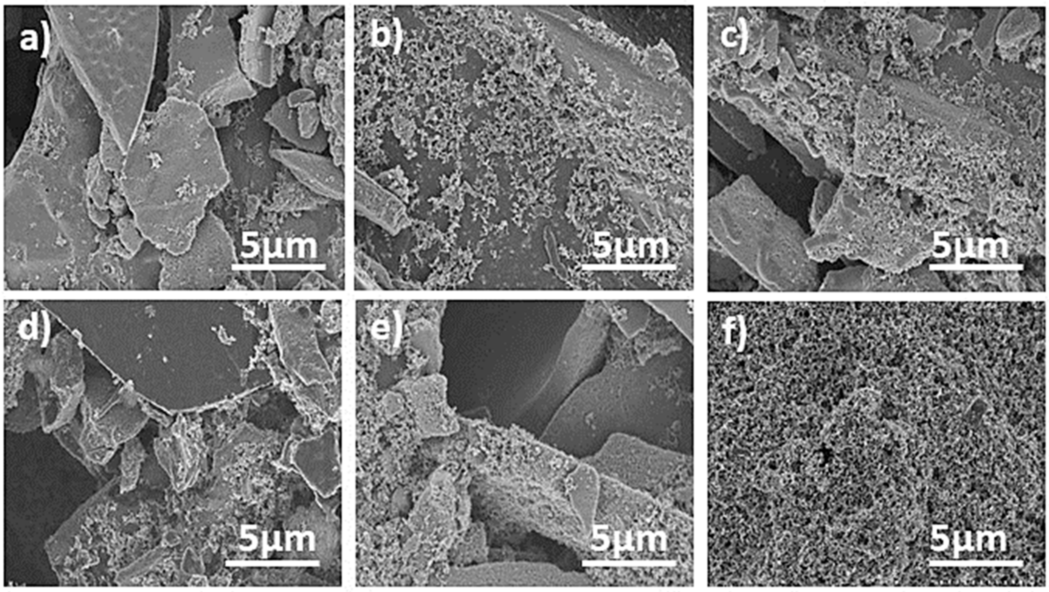

Figure S1. Field emission scanning electron microscope (FESEM) images of **(a)** AC **(b)** AC/RGO-5 **(c)** AC/RGO-10 **(d)** AC/RGO-15 **(e)** AC/RGO-20 and **(f)** RGO electrodes.

1.2. Thermogravimetric Analysis (TGA)

To understand the stability of GO and RGO, thermogravimetric analysis (TGA) was done under nitrogen and GO in Figure S2 shows a significant weight loss (6.5%) at 179.6 °C while little weight loss (1.2%) is observed for RGO at this temperature. There is a probable evaporation of adsorbed water molecules at temperature below 180°, leading to instability in the nature of GO. The significant weight loss between 179–300 °C is probably as a result of the decomposition of oxygenated functional groups (OFGs) to H_2O, CO_2 and CO gases [1]. The TGA of RGO shows a continuous weight loss with a relatively slow rate, revealing a higher stability compared to GO.

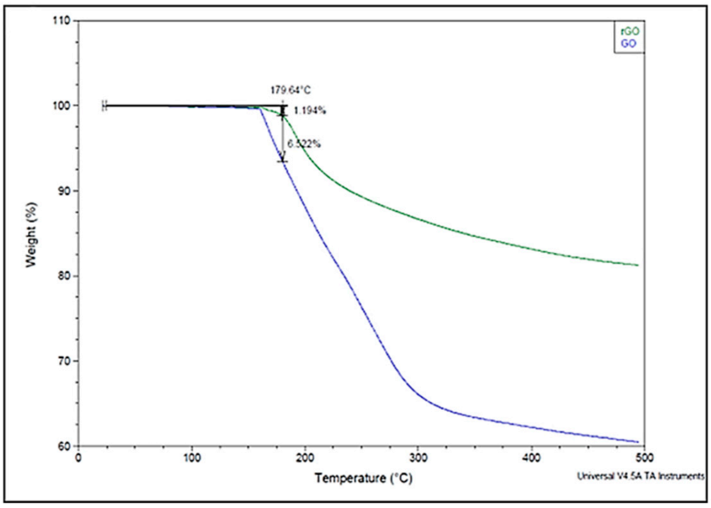

Figure S2. Thermogravimetric curves of GO and RGO.

1.3. X-ray Photon Spectroscopy (XPS)

Figure S3a,b shows the deconvoluted XPS spectra of GO and RGO respectively. Binding energies of 284.5, 285.8, 287 and 288 eV present in the distribution of the peaks corresponded to C=C, C-O, C=O and -COO- respectively [2]. After chemical reduction of GO, there is peak reduction in the binding energies of the oxygenated functional groups of RGO as made obvious in Figure S3b.

Figure S3c shows the XPS spectra of GO and RGO. From the spectra, it is obvious that the peak intensity of O element present in RGO decreased having undergone chemical reduction on comparison with that of GO. After chemical reduction, the residual oxygen content of the total element is 23% indicating that some oxygen containing functional groups are still present.

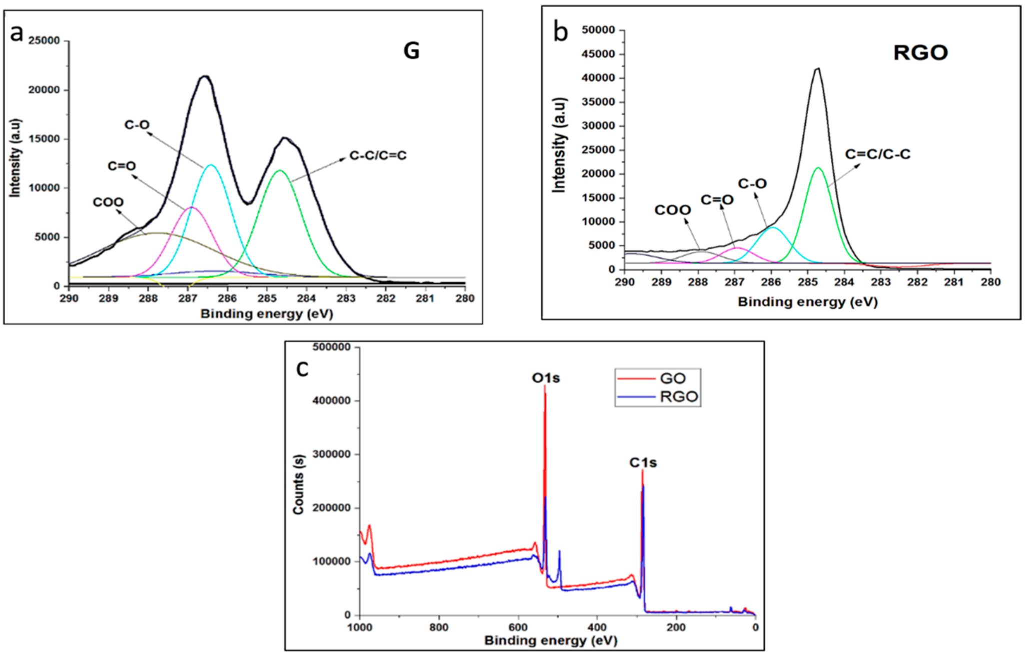

Figure S3. Deconvoluted XPS spectra of C1s of **(a)** and **(b)** GO and RGO respectively **(c)** XPS whole survey of pristine GO and RGO.

1.4. UV-Vis Spectrophotometry

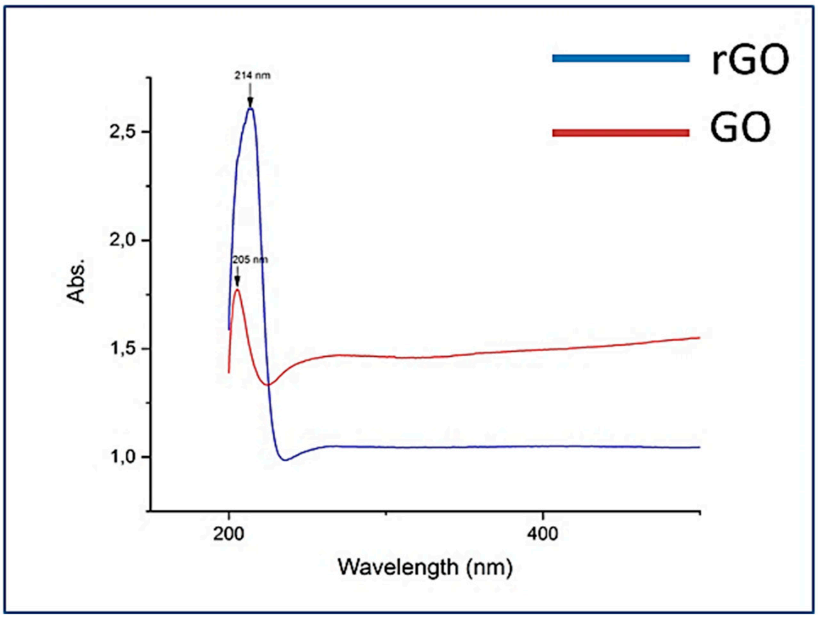

Figure S4. UV-Vis absorption spectroscopy shift of GO and RGO.

1.5. Electrochemical Property

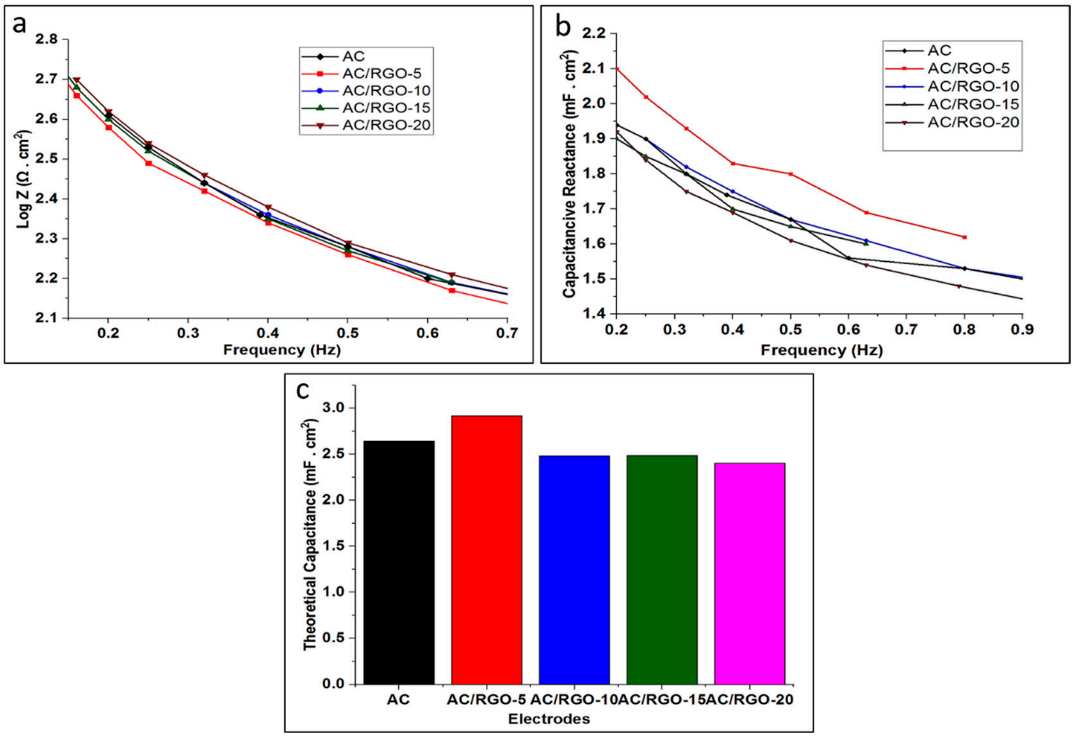

Figure S5. (a) Bode **(b)** Capacitive reactance plot of AC and AC/RGO-x as a function of frequency and **(c)** Histogram plot of theoretical capacitance of AC and AC/RGO-x from equivalent circuit fitting.

1.6. Capacitive Deionization Measurement

Table S1 presents the formulation for the solid electrodes used in this experiment.

Table S1. Solid electrode composition.

Copyright: © 2021 by the authors. Licensee MDPI, Basel, Switzerland. This article is an open access article distributed under the terms and conditions of the Creative Commons Attribution (CC BY) license (http://creativecommons.org/licenses/by/4.0/).

**Electrode material**	**AC (g)**	**PVDF (g)**	**CB (g)**	**RGO (g)**	**NMP (mL)**
AC	3.20	0.4	0.4	0.00	25
AC/RGO-5	2.64	0.4	0.4	0.16	25
AC/RGO-10	2.48	0.4	0.4	0.32	25
AC/RGO-15	2.32	0.4	0.4	0.48	25
AC/RGO-20	2.16	0.4	0.4	0.64	25

References

1. Malas, A., Das, C.K., Das, A. and Heinrich, G. Development of expanded graphite filled natural rubber vulcanizates in presence and absence of carbon black: Mechanical, thermal and morphological properties. *Mater. Des.* **2012**, *39*, 410–417.
2. Beidou G, Qian L, Erdan C, Hewei, Z., Liang, F., Jian Ru, G. Controllable N-doping of graphene. *Nano Lett.* **2010**, *10*, 4975–4980.
